# Supplementary material for: Activation of Humoral Immunity during the Pathogenesis of Experimental Chronic Lung Allograft Dysfunction
Source: Int J Mol Sci. 2022 Jul 23;23(15):8111. doi: 10.3390/ijms23158111 (PMC9331602; doi:10.3390/ijms23158111)
Supplement: Supplementary file 1 [file ijms-23-08111-s001.zip › Figure S1.pdf]

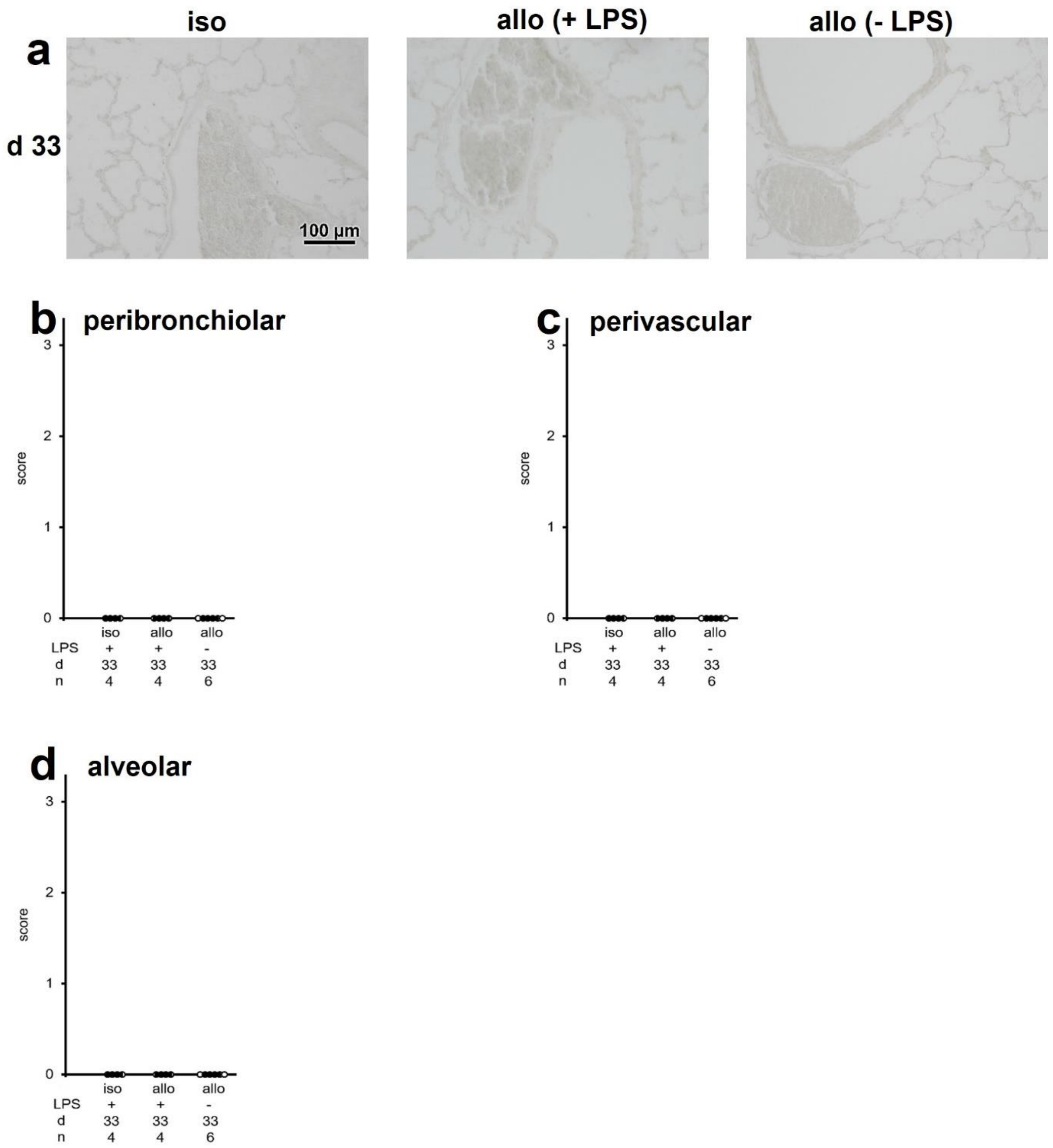

Figure S1: Infiltration of right native lungs by B cells at day 33 after left lung transplantation. Monoclonal antibody OX33 was used to detect B cells by immunohistochemistry on paraffin sections of right native lungs after isogeneic (iso) and allogeneic (allo) left lung transplantation. Right native lungs were investigated on postoperative day 33 (d 33), 5 days after treatment with LPS, respectively. Immunopositive cells are stained in brown, cell nuclei are lightly counterstained with hemalum (a). The intensity of B cell infiltration in peribronchiolar (b) perivascular (c) and alveolar (d) regions was evaluated using a scoring system ranging from 0 for virtually no B cell infiltrates to 3, the strongest infiltrates observed. Data are presented as individual data points, bar represents median, whiskers encompass the 25th to 75th percentile.
