# Supplementary material for: Activation of Humoral Immunity during the Pathogenesis of Experimental Chronic Lung Allograft Dysfunction
Source: Int J Mol Sci. 2022 Jul 23;23(15):8111. doi: 10.3390/ijms23158111 (PMC9331602; doi:10.3390/ijms23158111)
Supplement: Supplementary file 1 [file ijms-23-08111-s001.zip › Figure S2.pdf]

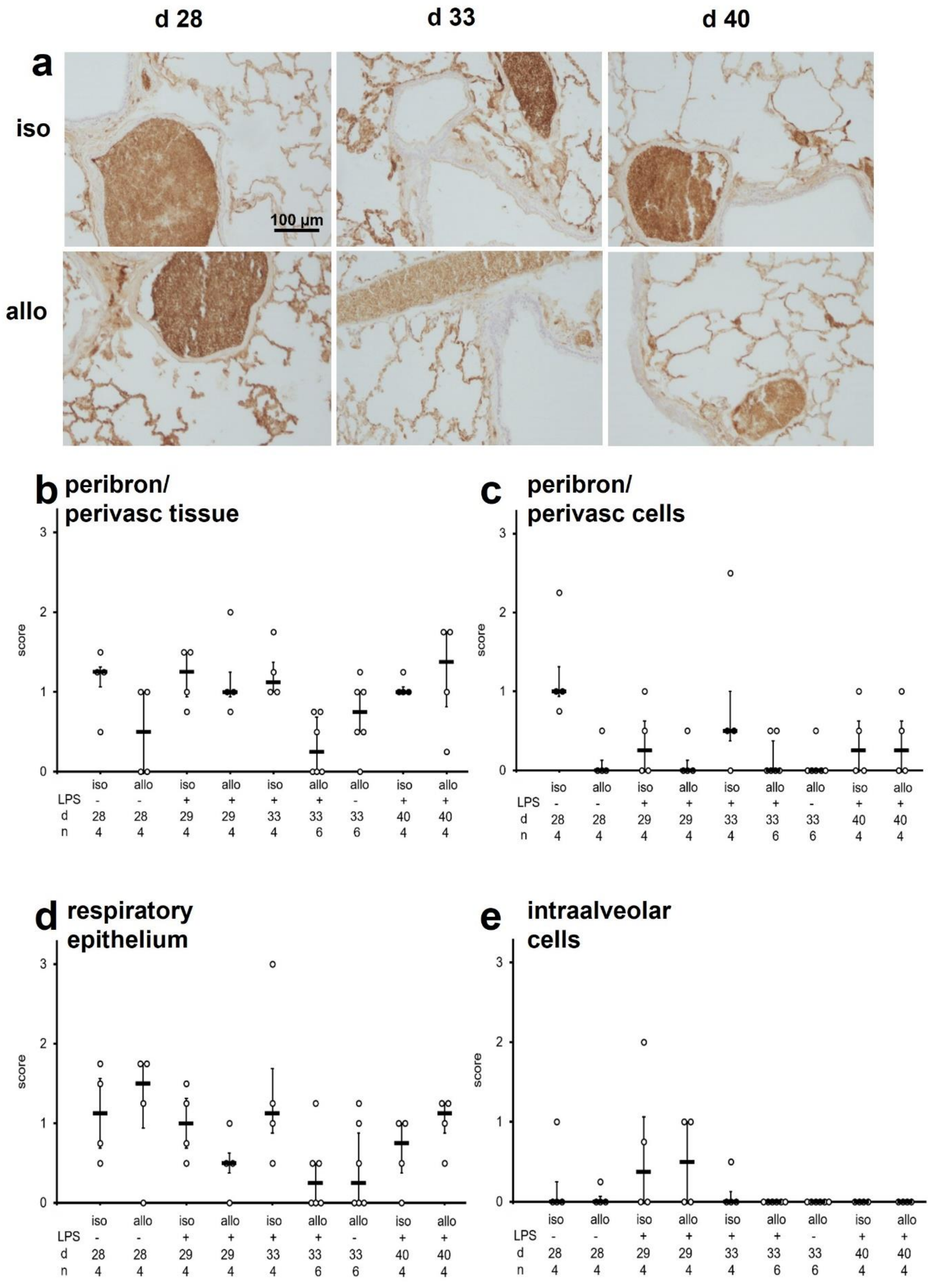

Figure S2: Accumulation of immunoglobulins (Ig) in right native lung tissue from left lung transplant recipients. Antisera directed to rat Ig were applied to paraffin sections of right native lungs after isogeneic (iso) and allogeneic (allo) left lung transplantation. Right native lungs were investigated on postoperative day 28 (d 28) before intratracheal instillation of lipopolysaccharide (LPS) or phosphate buffered saline (PBS) as well as on postoperative days 29 (d 29), 33 (d 33) and 40 (d 40), 1, 5 and 12 days after treatment with LPS, respectively.

Immunopositive regions and cells are stained in brown, cell nuclei are lightly counterstained with hemalum (a). The intensity of Ig deposits in peribronchiolar / perivascular (peribron/perivasc) tissue (b), the abundance of Ig-positive cells in peribronchiolar / perivascular regions (c), the intensity of Ig deposits on the luminal surface of the respiratory epithelium (d), and the abundance of Ig-positive cells in alveoli (e) were evaluated using a scoring system ranging from 0 for virtually no immunoreactivity to 3, the strongest immunoreactivity observed. Data are presented as individual data points, bar represents median, whiskers encompass the 25th to 75th percentile.
