# Supplementary material for: Activation of Humoral Immunity during the Pathogenesis of Experimental Chronic Lung Allograft Dysfunction
Source: Int J Mol Sci. 2022 Jul 23;23(15):8111. doi: 10.3390/ijms23158111 (PMC9331602; doi:10.3390/ijms23158111)
Supplement: Supplementary file 1 [file ijms-23-08111-s001.zip › Figure S3.pdf]

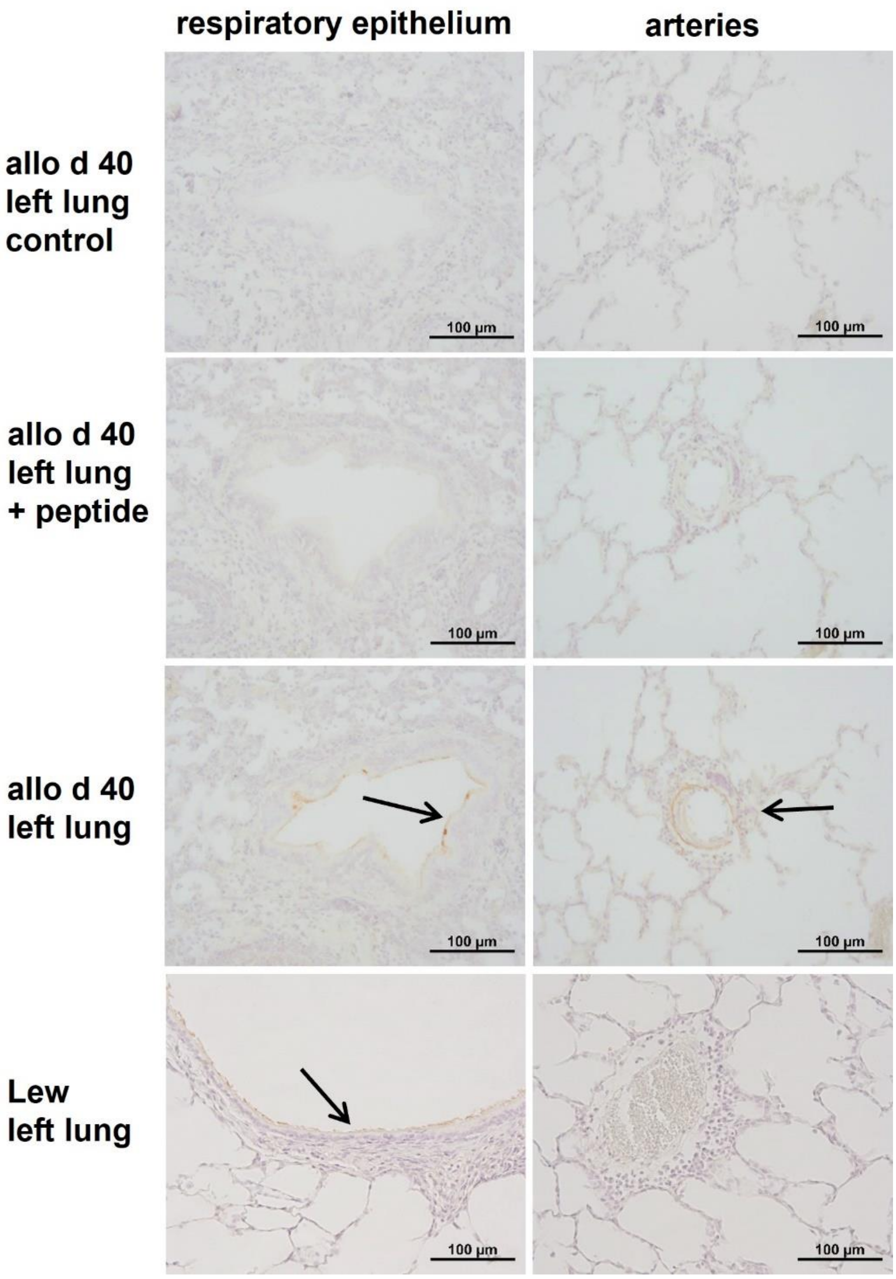

Figure S3: Controls of C4d immunohistochemistry. C4d was detected by immunohistochemistry on paraffin sections of pulmonary allografts (allo) on postoperative day 40, twelve days after treatment with LPS, respectively, and of native left lungs from untreated Lewis rats. Immunopositive regions are stained in brown (arrows), cell nuclei are lightly counterstained with hemalum. Examples of sections of d 40 allografts are depicted. In the section labelled control, the primary antibody was omitted and virtually no immunoreactivity was detected. Also, in the presence of the peptide that was used to raise the anti-C4d antiserum virtually no immunoreactivity was visible. A weak C4d immunoreactivity was seen on the luminal surface of the respiratory epithelium of native left lungs from untreated Lewis rats.
