# Supplementary material for: Activation of Humoral Immunity during the Pathogenesis of Experimental Chronic Lung Allograft Dysfunction
Source: Int J Mol Sci. 2022 Jul 23;23(15):8111. doi: 10.3390/ijms23158111 (PMC9331602; doi:10.3390/ijms23158111)
Supplement: Supplementary file 1 [file ijms-23-08111-s001.zip › Figure S4.pdf]

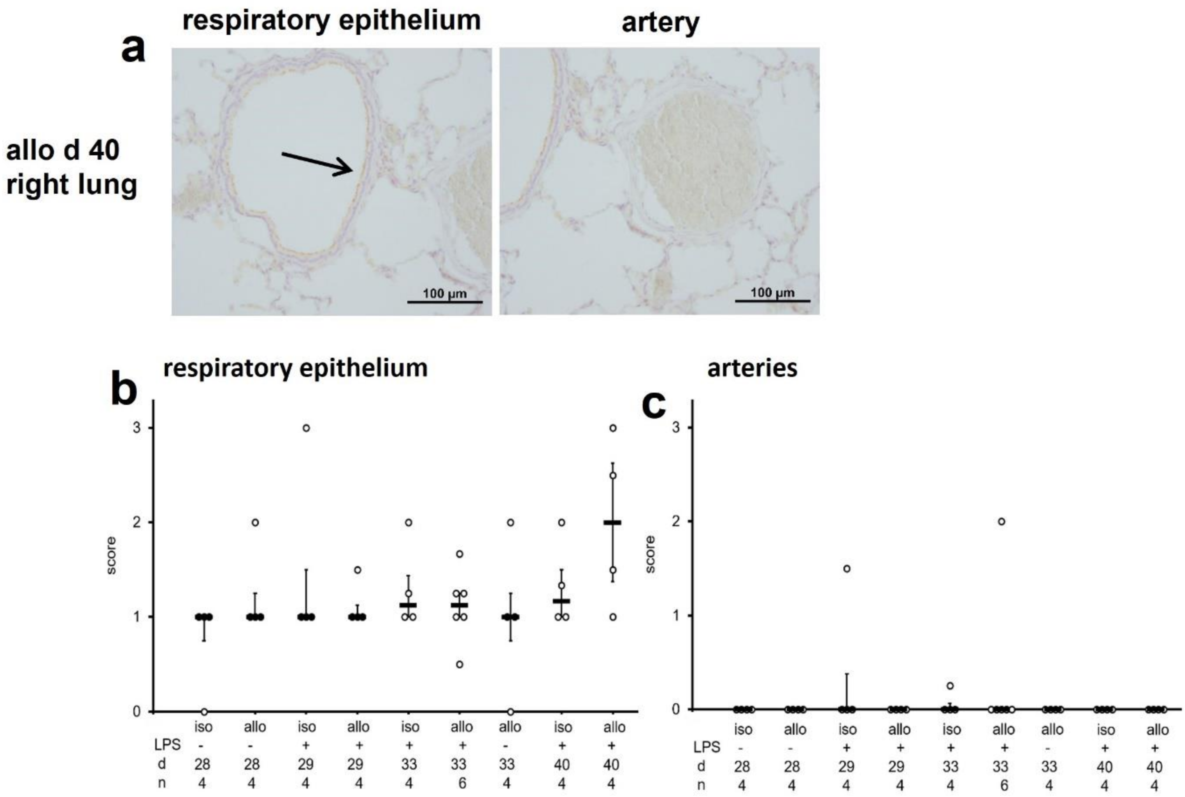

Figure S4: Deposition of C4d in right native lung tissue from left lung transplant recipients. C4d was detected by immunohistochemistry on paraffin sections of right native lungs after isogeneic (iso) and allogeneic (allo) left lung transplantation. Right native lungs were investigated on postoperative day 28 (d 28) before intratracheal instillation of lipopolysaccharide (LPS) as well as on postoperative days 29 (d 29), 33 (d 33) and 40 (d 40), 1, 5 and 12 days after treatment with LPS, respectively. Immunopositive regions are stained in brown, cell nuclei are lightly counterstained with hemalum. Examples of sections of d 40 allografts are depicted (a). In the section labelled control, the primary antibody was omitted (a). Arrow indicate the slight C4d deposit on the respiratory epithelium. The intensity of the C4d immunoreactivity on the respiratory epithelium (b) and in the arterial wall (c) was evaluated using a scoring system ranging from 0 for virtually no immunoreactivity to 3, the strongest immunoreactivity observed. Data are presented as individual data points, bar represents median, whiskers encompass the 25th to 75th percentile.
